# Adaptation of Cost Analysis Studies in Practice Guidelines

**DOI:** 10.1097/MD.0000000000002365

**Published:** 2015-12-31

**Authors:** Fainareti N. Zervou, Ioannis M. Zacharioudakis, Elina Eleftheria Pliakos, Christos A. Grigoras, Panayiotis D. Ziakas, Eleftherios Mylonakis

**Affiliations:** From the Infectious Diseases Division, Warren Alpert Medical School of Brown University, Providence, RI 02903.

## Abstract

Clinical guidelines play a central role in day-to-day practice. We assessed the degree of incorporation of cost analyses to guidelines and identified modifiable characteristics that could affect the level of incorporation.

We selected the 100 most cited guidelines listed on the National Guideline Clearinghouse (http://www.guideline.gov) and determined the number of guidelines that used cost analyses in their reasoning and the overall percentage of incorporation of relevant cost analyses available in PubMed. Differences between medical specialties were also studied. Then, we performed a case–control study using incorporated and not incorporated cost analyses after 1:1 matching by study subject and compared them by the Consolidated Health Economic Evaluation Reporting Standards (CHEERS) statement requirements and other criteria.

We found that 57% of guidelines do not use any cost justification. Guidelines incorporate a weighted average of 6.0% (95% confidence interval [CI] 4.3–7.9) among 3396 available cost analyses, with cardiology and infectious diseases guidelines incorporating 10.8% (95% CI 5.3–18.1) and 9.9% (95% CI 3.9– 18.2), respectively, and hematology/oncology and urology guidelines incorporating 4.5% (95% CI 1.6–8.6) and 1.6% (95% CI 0.4–3.5), respectively. Based on the CHEERS requirements, the mean number of items reported by the 148 incorporated cost analyses was 18.6 (SD = 3.7), a small but significant difference over controls (17.8 items; *P* = 0.02). Included analyses were also more likely to directly relate cost reductions to healthcare outcomes (92.6% vs 81.1%, *P* = 0.004) and declare the funding source (72.3% vs 53.4%, *P* < 0.001), while similar number of cases and controls reported a noncommercial funding source (71% vs 72.7%; *P* = 0.8).

Guidelines remain an underused mechanism for the cost-effective allocation of available resources and a minority of practice guidelines incorporates cost analyses utilizing only 6% of the available cost analyses. Fulfilling the CHEERS requirements, directly relating costs with healthcare outcomes and transparently declaring the funding source seem to be valued by guideline-writing committees.

## Abstract 2

Supplemental Digital Content is available in the text

## INTRODUCTION

In 2011, the total US health expenditures reached $2.7 trillion, which corresponded to 17.9% of the gross domestic product.^1^ In an effort to contain the overall cost, insurers move toward global budgets,^2^ and accountable care organization (ACO) models.^3^ However, it has been shown that simply providing physicians with cost data can reduce the overall cost of practice.^4^ Toward the goal of reducing medical expenses while maintaining the quality of healthcare practice, cost analysis studies are increasingly recognized as a fundamental tool as they provide estimations for the value of efficiency of alternative interventions.

Aim of this study was first to evaluate the incorporation of cost analyses in current guidelines both in absolute numbers and in relation to the available published studies for each topic and identify differences between medical specialties. Second, we aimed to determine modifiable characteristics of cost analyses that could make them more likely to be incorporated in practice guidelines.

## METHODS

### Incorporation of Cost Analyses in Current Guidelines

#### Selection of Guidelines

Between 24th and 28th of February 2014, we searched the National Guideline Clearinghouse (NGC, http://www.guideline.gov), and extracted all available guidelines. NGC is a database of evidence-based clinical practice guidelines maintained by the Agency for Healthcare Research and Quality of the US Department of Health and Human Services. NGC uses the definition of the Institute of Medicine for clinical practices guidelines that is “statements that include recommendations intended to optimize patient care that are informed by a systematic review of evidence and an assessment of the benefits and harms of alternative care options”^5^ and requires that “the guideline must have been developed, reviewed, or revised within the past 5 years” (http://www.guideline.gov).

From the extracted guidelines, we selected the 100 most cited, in an effort to: identify those that are more influential, include guidelines from different fields of medicine, and select guidelines that are more likely to relate to fields of medicine with active research in new therapeutic and diagnostic options; the appropriate use of which is a key factor in containing healthcare costs.

To count the citations, we used the Web of Science because it outperforms other databases in tracking citations from group-authored articles.^6^ We performed the evaluation of the number of citations within 1 work week, in order to minimize possible alteration of results by the addition of any new citations during the search period. In cases that the current guideline was an update of a previous version, we added the citations of all available updates, eliminating potential bias against recently published guidelines.

#### Detection of Incorporated Cost Analyses

A cost analysis study was defined as any “comparative analysis of alternative courses of action in terms of both their costs and consequences.”^7^ One author (FNZ) identified the 100 most cited guidelines. Two authors (EEP and IMZ) further scrutinized each of the 100 most cited guidelines to locate cost analysis studies. The search was done by examining the references of each guideline, and by scanning the text for the keywords “cost,” “economic,” and “value.” In this evaluation, we included systematic reviews of economic studies that were referenced in the guidelines. However, cost analysis studies that were cited in the guideline for a reason other than the implication of cost were excluded from the calculations.

#### Identification of Relevant Cost Analyses Studies

Two authors (IMZ and EEP) searched the PubMed database up to July 15, 2014 to identify cost analyses relevant to the subject of each guideline. When guidelines reported the search terms used, we combined those terms with the terms (cost OR economic^∗^) for our database search. If the search strategy was not indicated, then we used either a combination of topics covered in each guideline or Mesh terms of each included topic. In a second step, we confirmed the relevance of the subject of retrieved cost analysis studies with the guideline and excluded editorials, letters to the editor, and studies that, although relevant to the topic, could not have been included in the guideline for other reasons (such as cost analysis studies of hypothetical vaccines, cost analysis studies of treatment options no longer considered efficacious, etc.). Finally, we examined if cost analyses referenced in guidelines were retrieved from our search. We a priori set a goal of retrieval of 85% to characterize our strategy as successful.

### Determination of Characteristics That Differed Between Incorporated and Not Incorporated Cost Analyses: A Case–Control Study

#### Assigning Controls to Cases

Cases were defined as cost analyses incorporated (cited) into the guidelines. For each case, we assigned a control that matched the case by subject and was published before the corresponding guideline. To identify controls, we searched the related publications that were reported in PubMed for each case and scrutinized the articles retrieved from the broad search done for the corresponding guideline. If no relevant citations were found, the case was considered unique for that specific topic and was not matched. When more than 1 relevant citations were retrieved, the control was determined using a random sequence generator. In this part of the study, we excluded systematic reviews of economic studies.

#### Case–Control Study

Case and control studies were rated according to the requirements of the Consolidated Health Economic Evaluation Reporting Standards (CHEERS) statement; a 24-item guideline for the reporting of cost analyses.^8^ The fact that the CHEERS statement was published in 2013 ensured that all cases and controls included in our analysis were blinded to the requirements of the scale. In a pilot test, 2 authors (FNZ and IMZ) rated 300 cost analysis studies and concluded in a standardized way of evaluation of cost analysis studies. These authors, blind to the case–control allocation, rated studies and determined an overall score for each study (as the number of reported CHEERS items). The reproducibility of their results was estimated by the Cohen kappa statistic. We also examined differences in individual characteristics that may have contributed to inclusion or exclusion of a particular cost analysis from the guidelines. Additional variables analyzed included the year of publication, the impact factor of the publishing journal and whether the cost analysis study was industry-funded or not. Ethical approval was not necessary as this study was based on published data.

#### Statistical Analysis

A 1-way analysis of variance (ANOVA) was performed to examine the effect of year of publication on the probability of a cost analysis study to be included in a clinical guideline. In addition, we performed a 1-way ANOVA to investigate the effect of discipline on the probability of a cost analysis study to be included in a guideline, taking into consideration the 3 most cited guidelines per discipline. Categorical variables were expressed as frequency (%) and compared using the chi-squared test. Continuous variables were described as mean (standard deviation, SD) and compared using *t* test. The probability of using a cost-effectiveness analysis relative to the available cost-effectiveness articles on the subject was pooled for each discipline as a weighted proportion (with 95% random-effects confidence intervals [CIs]), as previously described.^9^ We used the Stata v13 software package (Stata Corporation, College Station, TX) for data analysis.

## RESULTS

**FIGURE 1:**
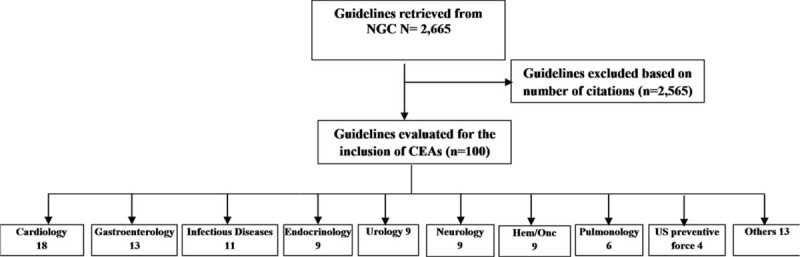
Selection process for guidelines included in our analysis.

We retrieved 2665 guidelines from the NGC (Fig. 1). The 100 most cited guidelines that were included in the final analysis had between 265 and 4797 citations. The majority of these guidelines were developed by medical associations based in the United States (78/100), while 18 were created by European medical associations and 4 through cooperation of US and European associations.

We found that 57/100 guidelines did not incorporate any cost analysis study, while, 17 included a single cost analysis and 26 included 2 or more. Among the 18 guidelines authored by a cardiology society, 10 (55.6%) included more than 2 cost analyses. Among the other medical societies that were represented by more than 5 guidelines in the total pool of 100 most cited, 50% (3/6) of the guidelines authored by a society of pulmonology, 38.5% (5/13) of gastroenterology, 36.4% (4/11) of infectious diseases, 12.5% (1/8) of urology, 11.1% (1/9) of hematology/oncology, 11.1% (1/9) of endocrinology, and 0% (0/9) of neurology had incorporated cost analyses in their reasoning. Also, none out of 4 (0%) guidelines authored by the US Preventive Services Task Force (USPSTF) included any cost analysis study (Table 1). The probability of a cost analysis study to be included in a clinical guideline was not affected by the publication year (F[11, 88] = 0.70, *P* = 0.738). Moreover, the discipline had no significant effect on the probability of a cost analysis study to be included in a guideline (F[7, 16] = 1.34, *P* = 0.294).

**TABLE 1:**
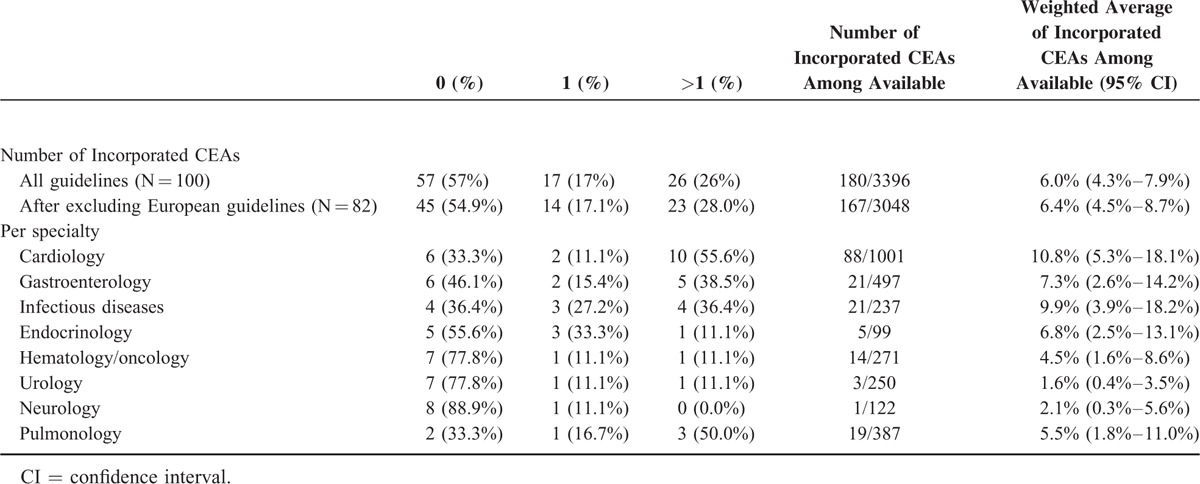
Number of Included Cost Analyses (CEAs) in Guidelines

The PubMed database search yielded 3396 cost analyses whose results had relevance to the subject of the guidelines (Supplemental Figure). Our search retrieved 90% of the cost analyses that were included in the guidelines and was considered efficient (surpassing our initial threshold of 85%). Importantly, only 6.0% (95% CI 4.3–7.9, τ^2^ = 0.083) of the available cost analyses were incorporated into the relevant guidelines (Table 1). After excluding guidelines authored exclusively by European medical associations, the estimation did not change (6.4%, 95% CI 4.5–8.7, τ^2^ = 0.092).

The guidelines authored by cardiology societies had included 10.8% (5.3–18.1%, τ^2^ = 0.159) of the available cost analyses. The corresponding number for infectious diseases societies was 9.9% (3.9–18.2%, τ^2^ = 0.105), for gastrointestinal 7.3% (2.6–14.2%, τ^2^ = 0.103), for endocrinology 6.8% (2.5–13.1%, τ^2^ = 0.011), for pulmonology 5.5% (1.8–11.0%, τ^2^ = 0.037), for hematology/oncology 4.5% (1.6–8.6%, τ^2^ = 0.014), for neurology 2.1% (0.3–5.6%, τ^2^ = 0.004), and for urology 1.6% (0.4–3.5%, τ^2^ = 0.000). The USPSTF did not use any out of the 248 relevant cost analyses.

A total of 148 pairs of cases and controls were included in the case–control study. The Cohen kappa for the CHEERS rating between the 2 authors was 0.89. The mean CHEERS score of cases was 18.6 (SD = 3.7) and was significantly higher than that of controls, 17.8 (SD = 4.2; *P* = 0.02; Table 2). Also, we examined differences between cases and controls in individual characteristics of the CHEERS statement. More specifically, the cost analyses included in the guidelines had healthcare outcomes in 92.6% as opposed to 81.1% of controls, and this difference was statistically significant (*P* = 0.004). Also, a significantly higher percentage of cases declared the funding source (72.3% vs 53.4%, *P* < 0.001). Across cases, 65.5% of the studies included a sensitivity analysis to examine the robustness of the results compared to 66.2% of controls (*P* = 0.9). The results of cases were reported as incremental ratios (additional cost per benefit unit) in 70.3% whereas that of controls in 60.8%, and this trend was not statistically significant (*P* = 0.09). Similar percentage of cases (68.2%) and controls (63.5%) reported the year of estimated costs (*P* = 0.39) and the yearly rate of discount for costs (56.8% vs 50%, *P* = 0.13). Finally, the efficacy of different strategies was based on the results of a single trial in 49.3% of cases and 48.6% of controls (*P* = 0.9) (the rest 50.7% and 51.4%, respectively, used literature synthesis to determine effectiveness).

**TABLE 2:**
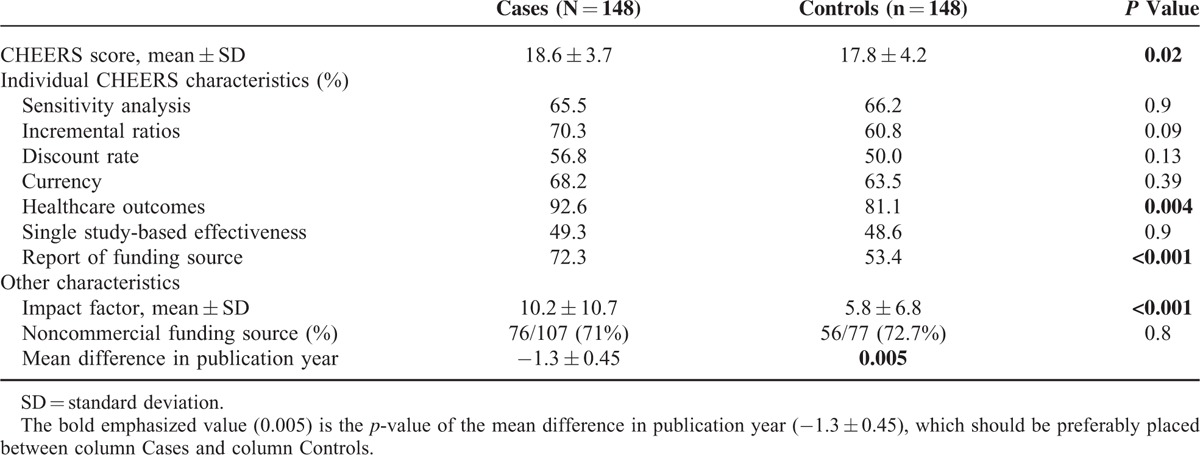
Results of the Case–Control Study

Overall, among the 107 cases and the 77 controls that reported the source of funding, 71% and 72.7%, respectively, had a noncommercial funding source (*P* = 0.8). The mean impact factor of the journal where the cases were published was 10.2 (SD = 10.7), significantly higher than the mean 5.8 of controls (SD = 6.8; *P* < 0.001). Finally, cost analyses that were included in the guidelines were published on average 1.3 years earlier than those not included (mean difference cases and controls −1.3, *P* = 0.005).

## DISCUSSION

Optimal allocation of healthcare resources depends on cost-effective clinical practice and clinical practice guidelines provide physicians and patients with valuable graded summary of evidence. Nevertheless, we found that the majority (57%) of the most highly cited clinical practice guidelines did not include any cost analysis. Similarly, the overall estimated percentage of inclusion of the relevant published cost analyses was only 6%, indicating that low adoption is not because of lack of relevant studies. We found significant differences between specialties. The characteristics that differentiated the included analyses were the higher overall quality of reporting, the association of estimated costs with healthcare outcomes, and the transparency in reporting the receipt of funding regardless of the funding source itself.

Previous studies performed in the 1990s noted that a low percentage of practice guidelines incorporated cost data.^10^ Nowadays, the rising healthcare costs and the financial burden imposed to patients,^11,12^ has led a high percentage of guideline committees to state in their methodological statements that they consider cost when developing recommendations.^13^ However, we found that this consideration has not led to higher inclusion of cost justification in current practice guidelines. In an effort to further clarify the reasoning behind our findings, we examined the availability of published cost evidence for each topic, differences between medical specialties, and, most importantly, differences between the included and not included studies regarding modifiable characteristics of cost analyses.

Interestingly, as noted above, adoption of cost analyses in clinical practice guidelines varied significantly between medical specialties. Two examples were the guidelines authored by societies of cardiology and hematology/oncology. Even though both heart diseases and cancer are among the 5 most costly conditions, with total medical expenditures of $74.1 and $71.6 billion, respectively; in 2012,^14^ these 2 specialties had a significant difference in policies regarding cost analyses. Specifically, the societies of cardiology systematically included cost analyses in 55.6% of their guidelines, incorporating a total of 10.8% of the relevant published studies. On the other hand, only 11.1% of the guidelines authored by hematology/oncology societies incorporated cost analysis studies. A relevant point is that the American Heart Association and American College of Cardiology recently published a statement regarding the systematic inclusion of resource utilization data in clinical practice guidelines. A grading system of the level of value and the robustness of available evidence will also be used, paralleling those for scientific level of evidence.^15^ Another finding that should be noted is the zero incorporation of cost analyses in guidelines authored by the USPSTF; a panel of experts that make evidence-based recommendations about preventive strategies. This was the case even for guidelines on preventive strategies of questionable efficacy where the task force was unable to determine the balances of benefits and harms of the service.^16^ The implication of this finding is specifically important as we move toward ACOs,^3^ with emphasis on primary care and a significantly more important role of preventive care. Data regarding cost benefit of preventive care will be critical both in convincing patients to follow guidelines and to get the government and other payers to appropriately reimburse cost-effective medical practice.

Unlike studies for clinical efficacy that have a gold standard when determining their level of evidence, cost analysis studies do not come with exact prespecified gold standards for conduction and reporting, creating difficulties in the interpretation and translation of their results into applicable changes in daily practice. The recent publication of the CHEERS guidelines,^8^ enabled us to quantify the differences between cases and controls and formulate guidance for the researchers who work on cost analyses regarding the key elements that may make cost analyses to be influential. The sub-analysis of individual study characteristics showed that it is significant for the cost arm of the cost analytic studies to be directly related to healthcare outcomes (eg, deaths or infections averted, quality of life gained). Another important characteristic of cases was the reporting of funding. It is reasonable to assume that there is a fear of potential bias for more favorable outcomes in studies that do not declare funding sources. Interestingly, the comparison regarding the source of funding yielded insignificant results, indicating that similar percentage of cases and controls were industry-funded. Cost analyses sponsored by industry have been reported to have lower quality and more favorable results.^17^ However, in our analysis, it was shown that the overall high quality of reporting and the transparency of the funding source, rather than the funding source itself, influenced significantly the adaptation of a cost analysis in clinical guidelines.

Also, in our analysis, the included cost analyses were published 1 year earlier than controls. This can be attributed to the long time needed for the collection of evidence, validation of guidelines, and consensus of experts regarding the formulated recommendations. This observation along with the need for rapid adaptation of the conclusions of cost analyses suggests the need for assessment of cost studies independently from the formulation of clinical practice guidelines giving the potential for rapid update of economic suggestions.

A notable element of this study is that the guidelines that were included in our analysis were retrieved from the NGC with the vast majority being developed by medical associations in the United States. Although this resource may include all guidelines, provided that they comply with the definition of the Institute of Medicine and are developed under the auspices of medical specialty association or governmental agency, the generalizability of our conclusions to guidelines formulated by European societies is lower. However, in our study design, we decided to focus on guidelines listed by the NGC because the “single payer model” of European healthcare system might provide other opportunities for the adaptation of cost analyses, while in the United States, the incorporation of cost analyses in clinical guidelines seems to play a unique and central role. Investigating the parameters that influence the incorporation of cost analyses in clinical guidelines in different countries around the world could provide additional information and it is a field which justifies further research.

## CONCLUSIONS

In conclusion, the inclusion of relevant cost analyses was 6%, with a significant variation between different specialties. We identified a series of parameters that make a cost analysis more likely to influence medical practice, including the report of funding source and the direct association of cost changes to patients’ health outcomes. Without a central mandate to include a cost analysis as part of guidelines, adaptation will most likely continue to remain low.

## Supplementary Material

Supplemental Digital Content
