# Supplementary figures and images for: Adaptation of Cost Analysis Studies in Practice Guidelines
Source: Medicine (Baltimore). 2015 Dec 31;94(52):e2365. doi: 10.1097/MD.0000000000002365 (PMC5291618; doi:10.1097/MD.0000000000002365)

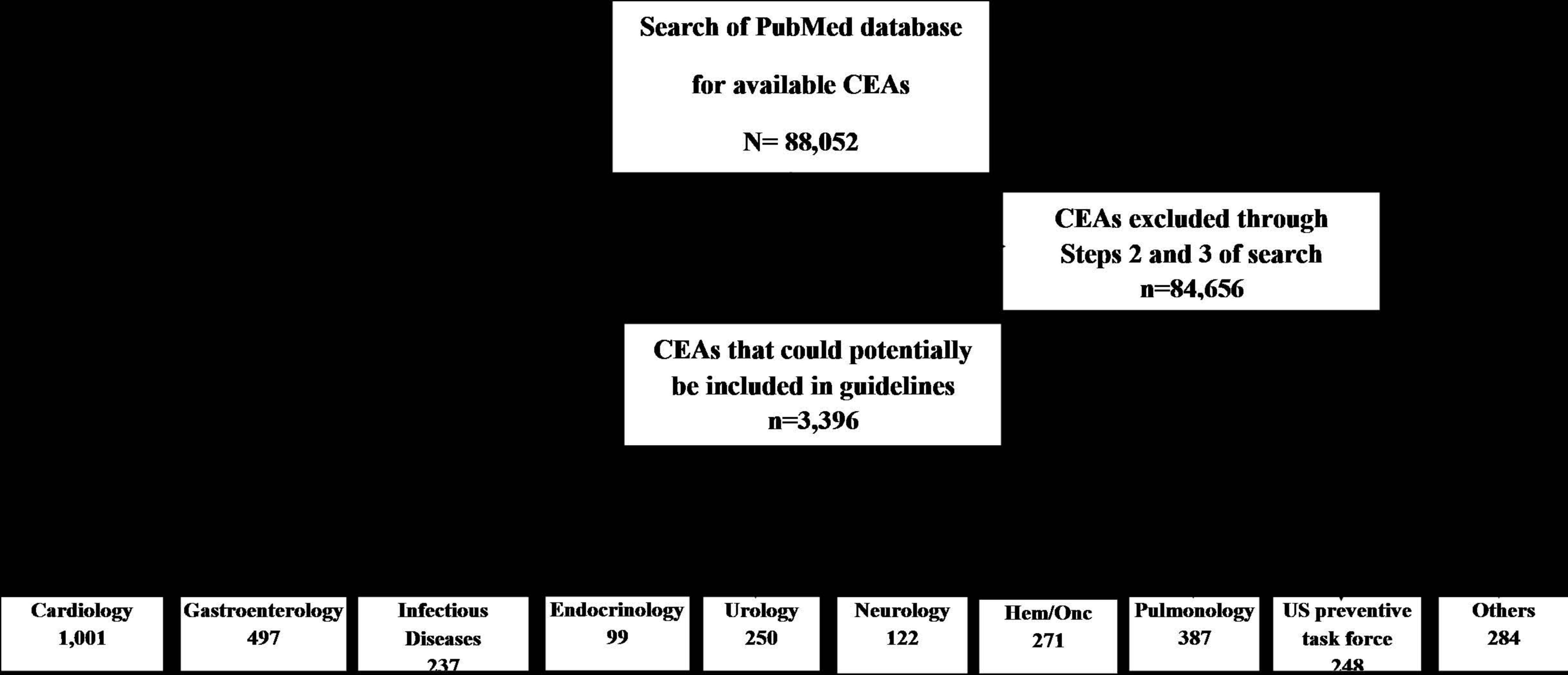

Supplement: Supplemental Digital Content [file medi-94-e2365-s001.pdf]
